# Unsparing self-critique strengthens the field, but Bailey et al. overstate the ‘problems with delay discounting’

**DOI:** 10.1017/S0033291721005286

**Published:** 2023-03

**Authors:** Jeffrey S. Stein, James MacKillop, Samuel M. McClure, Warren K. Bickel

**Affiliations:** 1 Fralin Biomedical Research Institute at VTC, Virginia Tech, Roanoke, VA, USA; 2 Department of Psychiatry and Behavioural Neurosciences, McMaster University, Hamilton, ON, Canada; 3 Department of Psychology, Arizona State University, Tempe, AZ, USA

We agree with a fundamental premise of Bailey, Romeu, and Finn's (2021) recent paper; that is, understanding the limitations of the delay discounting (DD) paradigm will increase the value of research on intertemporal choice (ITC) in clinical disorders. To this agreement, however, we add two provisos. First, the significance of those limitations should be determined by systematic review and meta-analysis, not selective review or personal opinion. Bailey et al.'s narrative review reflects a bias for their position that DD measures have not been adequately validated and lack generalizability, without regard for evidence that may mitigate these concerns. Second, the strengths of the paradigm should be acknowledged in order to avoid, in the pursuit of progress, weakening the measures we seek to refine. In what follows, we highlight our agreements and disagreements with Bailey et al.'s three major concerns: convergent validity, divergent validity, and generalizability of DD measures.

## Convergent validity

Bailey et al. acknowledge the well-known observation that DD is largely uncorrelated with measures of impulsivity. While accurate, this view fails to characterize the state of the field. Impulsivity itself is a multifactorial construct showing little correlation among its putative forms (e.g. acting without forethought, response disinhibition, and DD). Decades of research have failed to identify common neurobiological mechanisms among these various ‘impulsivities’, and the effects of behavioral and pharmacological interventions vary across forms. For these and other reasons, some have called for rejecting the superordinate construct of impulsivity in favor of continued, rigorous inquiry of its various component measures (Strickland & Johnson, 2021). We agree with this perspective.

Bailey et al. outline additional concerns over convergent validity, arguing that DD has yet to show strong and replicable associations with other cognitive and personality measures. These authors also suggest that, in order to be considered an ‘important’ individual difference, DD should show incremental validity over these other measures when examining associations with substance use disorder (SUD) and other clinical disorders. We agree that DD has shown relatively modest associations with executive function and personality measures, but these findings are replicable at the behavioral and neurobiological levels. Such consistent, but limited, covariance with a variety of measures suggests not necessarily, as Bailey et al. argue, that DD is an unimportant individual difference, but rather that its determinants are multivariate.

Moreover, Bailey et al. disregard emerging evidence of incremental validity. Specifically, using machine-learning approaches to predict SUD, measures of DD outperform the Stroop task, Iowa Gambling task, Continuous Performance task, and other measures of attention, planning, and inhibitory control (e.g. Bickel, Moody, Eddy, & Franck, 2017). Nonetheless, we agree that the literature would benefit from additional studies examining incremental validity, particularly in predicting health behaviors beyond substance use.

## Divergent validity

Bailey et al. note concerns over divergent validity of the DD paradigm, citing evidence that elevated DD is robustly associated with not only SUD but various other psychiatric disorders, including depression, bipolar disorder, and schizophrenia (for a meta-analysis, see Amlung et al., 2019). Thus, as Bailey et al. argue, measures of DD may not differentially predict diagnostic status among patients with varied disorders. This remains an empirical question and more research should examine the specificity of such associations. However, some research provides emerging evidence of divergent validity. For example, SUD and comorbid mood and personality disorders are interactively associated with higher DD compared to SUD alone (e.g. Moody, Franck, & Bickel, 2016). Conversely, anorexia nervosa is associated with *lower* DD compared to healthy controls (for a meta-analysis, see Amlung et al., 2019). Thus, DD rates at opposing ends of a continuum are associated with divergent pathology.

More generally, we note that the well-documented comorbidity between SUD and other psychiatric disorders suggests transdiagnostic risk factors and etiologies. That DD is associated with a variety of clinical endpoints is not necessarily a cause for concern, but may instead be a strength of the paradigm and provides the opportunity to investigate a single decision-making process relevant to a broad range of disorders. However, further research is needed to understand the circumstances under which genetic, neurobiological, or environmental predispositions manifest in divergent phenotypes. Indeed, this pursuit is a focus of the NIH's Research Domain Criteria (RDoC), an area in which DD holds some promise (e.g. Levitt et al., 2022).

## Generalizability

Bailey et al. argue that little evidence suggests that DD is a generalizable measure of decision-making. In the process, these authors disregard evidence of reliable correlations in measures of discounting across different commodities (e.g. money, food, health, and drugs of abuse; for a systematic review, see Odum et al., 2020). Thus, contrary to the limited findings reviewed by Bailey et al., knowledge of how an individual discounts one delayed commodity provides information about how they are likely to discount other delayed commodities.

Given this correspondence, the majority of studies on DD preferentially examine discounting of monetary outcomes because, unlike non-monetary outcomes, money is universally valued, easily quantifiable, highly fungible, and its utility curve is approximately linear across a broad range of values. We recognize, however, that discounting of non-monetary, pathology-relevant commodities in some circumstances may provide incremental validity over the study of monetary outcomes (e.g. discounting of food in the study of obesity; for a meta-analysis, see Amlung, Petker, Jackson, Balodis, & MacKillop, 2016). As such, future research should continue to examine the relationship between non-monetary discounting, SUD, and other disorders. Moreover, choice arrangements that may more closely model real-world ITC should also be explored (Green & Myerson, 2019). For example, ‘cross-commodity’ discounting tasks arrange choices between qualitatively different immediate and delayed outcomes, which may characterize intertemporal tradeoffs evident in health-related decision-making (e.g. immediate food *v.* delayed weight loss in the study of obesity).

## Conclusions

In the era of the ‘replication crisis’, the links between DD and clinical disorders are notably robust. At the heart of Bailey et al.'s concerns is the gap between theoretical concepts (in this case, ITC) and their operational definitions (in this case, DD). Critically, ITC is multifaceted, including multiple dimensions of time orientation (past, present, and future), gains and losses, and same- or cross-commodity choice. Bailey et al. take issue with the fact the literature operationalizes ITC in only one way (DD), contending this provides an incomplete perspective. We do not disagree and likewise recommend a more fulsome study of the published varieties of ITC. Indeed, we have conducted empirical research mapping these less studied varieties. Fundamentally, however, promoting this more expansive perspective should not justify overstating the limitations of the robust and trenchant literature on DD in SUD and other disorders.
